# Responsiveness of Isokinetic Dynamometry in Patients with Osteoarthritis after Knee and Hip Arthroplasty: A Prospective Repeated-Measures Cohort Study

**DOI:** 10.3390/healthcare12030314

**Published:** 2024-01-25

**Authors:** Ferdinand Prüfer, Monika Pavlović, Špela Matko, Stefan Löfler, Michael J. Fischer, Nejc Šarabon, Vincent Grote

**Affiliations:** 1 Ludwig Boltzmann Institute for Rehabilitation Research, A-1100 Vienna, Austriamichael.fischer@reha-kitz.at (M.J.F.); vincent.grote@rehabilitation.lbg.ac.at (V.G.); 2 Faculty of Health Sciences, University of Ljubljana, SI-1000 Ljubljana, Slovenia; monika.pavlovic@zf.uni-lj.si; 3 Vamed Rehabilitation Center Kitzbühel, A-6370 Kitzbühel, Austria; 4 Faculty of Health Sciences, University of Primorska, SI-6310 Izola, Slovenia; 5 Innorenew CoE, SI-6310 Izola, Slovenia

**Keywords:** Arthroplasty, Replacement, Hip, Arthroplasty, Replacement, Knee, muscle strength, physical functional performance, rehabilitation, responsiveness

## Abstract

Functional assessments are crucial for the evaluation of rehabilitation after total knee (TKA) and hip (THA) arthroplasty. Muscle strength, a key determinant of physical function (PF), is often measured with isokinetic dynamometry (ID), which is considered the gold standard. However, studies lack evaluations of responsiveness—the ability to detect changes over time. This study aims to determine the responsiveness of ID in measuring PF in TKA and THA rehabilitation—is muscle strength a valid indicator for assessing improvement in rehabilitation processes? The pre- and post-surgery PF of 20 osteoarthritis patients (age 55–82) was assessed, using ID, performance-based and self-reported measures. Responsiveness was evaluated by comparing the observed relationship of changes in ID and PF scores with the a priori defined expected relationship of change scores. While the performance-based and self-reported measures showed significant improvements post-surgery (Cohen’s *d* [0.42, 1.05] *p <* 0.05), ID showed no significant differences. Moderate correlations were found between changes in some ID parameters and selected functional tests (*r ≈|*0.5*|*, *p* < 0.05). Responsiveness was solely found for the peak torque of knee extension at 180°/s on the operated side. Responsiveness is an often-overlooked psychometric property of outcome measurements. The findings suggest that ID may not be fully responsive to the construct of PF after TKA and THA, raising questions about its role and usefulness in this context and the need for more appropriate assessment methods.

## 1. Introduction

Lower limb muscle weakness is a common consequence following joint replacement surgery and can adversely affect functional outcomes [1,2]. Physical function (PF) is a key determinant of health-related quality of life in these patients. Therefore, improving PF is one of the major goals in rehabilitation after hip and knee replacement surgery. However, it is important to note that there is a wide range of variability in PF outcomes. Some patients achieve excellent outcomes and can return to work and their pre-operative activities, while others may continue to experience some pain and limitations in their PF [3]. Measuring PF before and after surgery can help identify patients who are at risk for poor outcomes. This information can be then used to develop individualized treatment plans aiming at PF and quality of life improvement. Functional assessments are considered key outcome measures in the evaluations of health and well-being in the elderly [4] as well as in rehabilitation [5]. Lacking unity in terminology, the terms functional status, physical ability, physical performance, physical function, or physical functioning are used synonymously in the literature and describe the same construct [5,6]. The construct of PF can be defined as the ability to perform basic and instrumental activities of daily living (activities of daily living—ADL and instrumental activities of daily living—IADL) in order to live independently [6,7]. It is a multidimensional concept consisting of conceptually related but distinct subdomains, e.g., lower limb function or muscle performance [5,8]. One of the key factors determining PF is muscle strength [9,10]. In a recent endeavor, Jiang et al. [11] sought to develop a model to identify structural dimensions that are most pertinent in assessing physical function among community-dwelling adults and identified muscle strength as one of the three explaining factors. Consequently, it is reasonable to presume that changes in PF will be reflected by corresponding changes in muscle strength. However, little to no research exists on the relationship between changes in muscle strength and changes in PF.

PF in patients undergoing total knee arthroplasty (TKA) and total hip arthroplasty (THA) is measured using a variety of patient-reported outcome measures (PROMs) and clinician-reported outcome measures (CROMs). PROMs commonly used in studies include the Western Ontario McMaster University Osteoarthritis Index (WOMAC) or the Knee injury and Osteoarthritis Outcome Score (KOOS) and Hip disability and Osteoarthritis Outcome Score (HOOS) [12,13]. The CROMs include the Timed Up and Go test (TUG), the Stair Climb Test (SCT), walking tests and lower limb muscle strength [7,9,12,13,14,15]. Muscle strength is considered one of the key determinants of health-related quality of life and PF in patients after TKA [16,17,18]. In physical rehabilitation, it is also frequently used for the selection, control and evaluation of treatment and the recovery process. Changes in the level of lower extremity muscle strength are used by researchers and clinicians to draw conclusions about the effectiveness of treatment and patients’ progress after TKA and THA [19,20,21,22,23,24,25]. As clinicians or researchers, the ability to depend on these scores is crucial. Consequently, there is a notable research interest in ensuring that the assessment of muscle strength in patients undergoing THA and TKA is conducted using testing methods that are valid, reliable and responsive.

Isokinetic dynamometry (ID) is considered the gold standard for the assessment of muscle strength [26,27]. In ID, one or more contractions of an isolated muscle group are performed while the dynamometer maintains a constant preset velocity by providing precise resistances throughout the contraction to ensure that each contraction is performed at a specific velocity [28]. A strong correlation between knee extension strength and functional performance indicates that enhancing quadriceps strength could be crucial in maximizing the overall success of TKA [29]. Isokinetic muscle performance after THA and TKA is commonly measured as maximal peak torque (PT) at a given velocity [30,31]. While ID has been shown to be a safe, valid and reliable method to assess muscle strength [27,32,33], studies lack evaluations of responsiveness in patients after THA and TKA.

Responsiveness is “the ability of an instrument to detect change over time in the construct to be measured” [34]. According to Guyatt et al. [35], responsiveness to change is a core characteristic of evaluation instruments designed to measure longitudinal change over time. The COnsensus-based Standards for the selection of health Measurement INstruments (COSMIN) group considers responsiveness to be part of validity and it is therefore assessed by evaluating the “expected relationships between changes on the instrument under study and changes on other instruments that measure similar or different constructs” [36]. While validity refers to the validity of a single score (estimated on the basis of one measurement), responsiveness refers to the validity of a change score (estimated on the basis of two measurements) [37]. Improving the patient state of health and well-being is the main goal of rehabilitation. Monitoring changes helps in the selection of appropriate treatment paths and can be used to evaluate the success or failure of therapy. De Vet et al. [37] even state that the assessment of change in patient condition is “often the most important objective of measurements in clinical practice and clinical and health research”. Despite its importance in health and rehabilitation research, responsiveness is an often-overlooked psychometric property of outcome measures.

To date, no efforts have been made to define the responsiveness of ID assessment in patients undergoing total hip or total knee replacement surgery. Moreover, the association between changes in muscle strength and changes in physical function remains inadequately explored. We hypothesize that changes in isokinetic muscle strength adequately reflect changes in physical function in patients undergoing TKA and THA. Therefore, the main aim of this study is to answer the following question: Is ID responsive in measuring PF in patients after TKA and THA?

## 2. Materials and Methods

### 2.1. Study Design and Participants

This prospective cohort study with a repeated-measures design was conducted in an outpatient rehabilitation setting at the Institute for Physical Medicine at Wiener Gesundheitsverbund Klinik Ottakring, Vienna, Austria, from April 2022 to January 2023, as part of the AMB-REMOB (Early Outpatient Remobilisation after total knee and hip arthroplasty) project [38].

**Figure 1:**
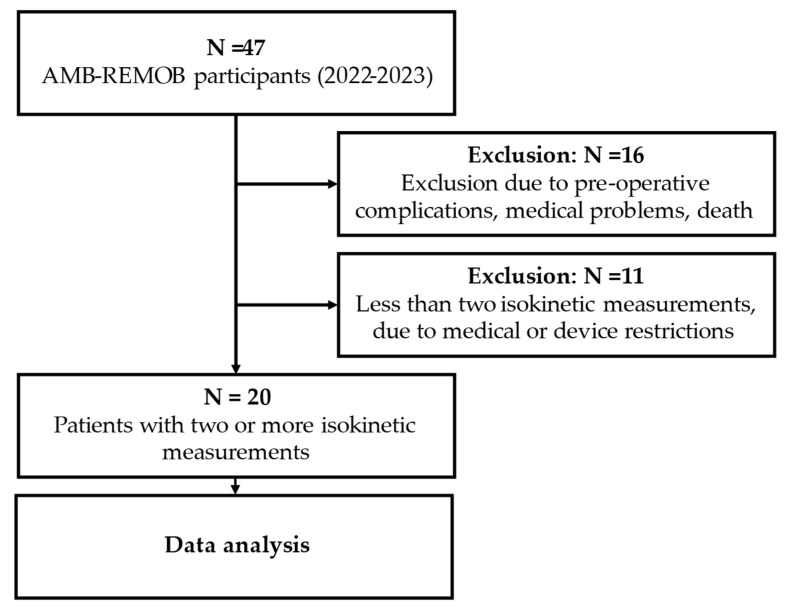
Flow diagram of study participants.

Patients were continuously recruited via participating surgical and orthopedic hospitals using a detailed letter-based approach. Individuals scheduled for TKA and THA surgeries between April 2022 and January 2023, due to chronic cartilage damage, were contacted. Out of the 173 patients initially contacted, 76 actively responded and conveyed their interest, ultimately resulting in the participation of 46 patients in the AMB-REMOB project. From this cohort, 20 individuals met the eligibility criteria and were included for analysis in the present study. Patients were included in the study if they had completed at least two isokinetic strength measurements (pre-surgery measurement and at least one post-surgery measurement) (Figure 1).

Data was collected at four measurement points, (T0) two weeks pre-surgery, (T1) two weeks post-surgery, (T2) six weeks post-surgery and (T3) ten weeks post-surgery. Due to clinical restrictions, isokinetic measurement at T1 was not authorized, making the measurements T0, T2 and T3 viable for evaluation in this study. In an effort to encompass the most participants possible and facilitate the observation of genuine changes, our approach involved assessing responsiveness by examining the alterations from T0 (pre-surgery) to T3 (10 weeks post-surgery). This decision was made to comprehensively evaluate responsiveness throughout the entire study duration, covering the phases of surgery and rehabilitation. The AMB-REMOB rehabilitation program lasted four weeks, starting two weeks post-surgery and consisting of underwater therapy, electrotherapy and three to four sessions per week, like the standard treatment in Austria (WHO Phase II).

Participants were 66.6 ± 7.8 years old and had a body mass index (BMI) of 30.0 ± 5.5. The sample consisted of 12 TKA and 8 THA patients. Further demographic characteristics of the subjects are documented in Table 1.

The AMB-REMOB study complies with the Declaration of Helsinki. The informed consent of all patients was collected. The clinical study was approved by the Ethics Committee of the City of Vienna. This study is registered in the German Register of Clinical Studies (DRKS00028152; UTN: U1111-1275-5181).

### 2.2. Outcome Measures

TUG, 10 m walk test (10 MWT), SCT, WOMAC, Health Assessment Questionnaire Disability Index (HAQ-DI) and ID measurements were conducted two weeks prior to surgery (T0) as well as six (T2) and ten weeks after surgery (T3). All measurements were organized and performed by trained kinesiologists and physical therapists at the outpatient rehabilitation institute. All measurements were consistently conducted during the same time window each day (8–11 a.m.). Measurement started with participants filling out the self-reported questionnaires, followed by performance-based measures. The sequence of functional tests was randomized for each patient at the initial time point (T0), by utilizing an online list randomizer (random.org). Notably, the order within subjects remained consistent throughout subsequent measurements. A brief 2 min general warm-up, comprising activities such as marching on the spot, heel digs, knee lifts and shoulder rolls was administered prior to functional testing. Additionally, each test was preceded by a specific warm-up involving a few trial repetitions. To prevent fatigue-induced effects, breaks of 1–2 min were implemented between repetitions and more extended breaks of 3–5 min were performed between different tests.

Muscle strengths of the knee extensors and flexors were assessed using isokinetic peak torque. In contrast to other methods like isometric testing, this method records the total muscle tension over the whole Range of Motion (ROM) [40], which enables a better evaluation of overall muscle performance. Isokinetic dynamometers are electromechanical resistance instruments containing a speed-controlling mechanism, which ensures a constant preset velocity while force is applied [41]. They automatically adapt to changes in muscle force during movement by providing counterforce, thus allowing for consistent maximum force generation throughout the entire ROM [41]. Multiple researchers have shown IDs to be a safe, valid and reliable method of muscle strength assessment in patients after THA and TKA [27,32,33].

Isokinetic measurements were performed using the Biodex System 4 Quick-Set™ dynamometer (Model 840-000, System 4 Quick Set, Biodex Medical Systems, Inc., Shirley, New York, NY, USA). Participants were seated with 90° hip flexion and 90° knee flexion. Starting with the uninvolved leg, the seating position for each patient and each leg was adjusted to allow maximum and comfortable knee extension and flexion, with the center of the knee aligned with the axis of rotation of the dynamometer’s lever arm. The measurement protocols provided by the Biodex system were employed for concentric isokinetic knee extension and flexion at two angular velocities: 60°/s with five repetitions and 180°/s with twenty repetitions. As knee extensors and flexors perform at various velocities during different activities and research has shown a decrease in maximum strength with an increase in angular velocities [42,43], we decided to measure isokinetic strength at two different angular velocities for a more comprehensive assessment of muscle performance. ROMs for each patient and limb weight correction were set in accordance with the Biodex user’s manual. The testing process began with the participants’ uninvolved legs positioned in maximum knee flexion. They were then instructed to perform knee extensions and flexions as forcefully and quickly as possible against the lever arm resistance, immediately reversing their movement at maximum extension/flexion. Before each formal measurement, patients were given practice repetitions to familiarize themselves with the system. Once patients felt comfortable and prepared, the actual measurements began, starting with the 60°/s five-repetition assessment. Following a one-minute break and getting accustomed to the new angular velocity, the second measurement at 180°/s with twenty repetitions was performed. Subsequent measurement of the uninvolved leg followed the measurement of the involved leg. During the measurements, patients were verbally encouraged by the instructors to perform at their maximum capacity, and the instructor also communicated the change in movement direction. Immediate performance feedback was displayed on the device’s integrated monitor. If at any point during the measurement process, patients exhibited pain or discomfort, the measurement was stopped immediately.

Maximum muscle strength was recorded as the average PT in Newton meters (Nm) of the highest three repetitions at each angular velocity, in each movement direction, resulting in eight different parameters (see Table 2).

The TUG is a performance-based PF measure, known to be valid, reliable, and responsive [44,45,46,47,48,49]. It has also been validated as a useful tool for the evaluation of PF after lower limb joint surgery [50]. TUG evaluates the time required for an individual to stand up from a regular armchair (with a seat height of 46 cm), walk a distance of 3 m, perform a turn, return to the chair and resume sitting [46]. Participants wear their normal footwear and use their usual walking aid if needed. The test starts with the participant seated with his back against the backrest, arms lying on the chair’s armrests and walking aid at hand. Given the command “go”, the participant was instructed to get up and walk “as fast and as safe as possible” to a cone placed on the floor 3 m from the chair, turn at the cone, return and sit down again. The time was measured with a stopwatch, starting when the participant’s buttocks left the chair and ending when they returned to the chair. Before the measurement, the participants performed a trial run. Three runs were performed, with short breaks in between. The fastest run was used for evaluation. To maintain consistency, the same chair was used in all measurements.

The SCT serves as a comprehensive assessment of lower body strength, balance and functional mobility [51]. It tests a person’s ability to ascend and descend a flight of stairs. An 11-step staircase with a handrail and a step height of 20 cm was used. Participants were instructed to ascend, immediately turn around and then descend the flight of stairs as quickly and as safely as possible. The timing, measured in seconds, started when one foot left the floor level and concluded when both feet returned to the floor level. To ensure participants’ safety, the use of the handrail as well as walking aids was permitted, if needed. For consistency, the same flight of stairs was used in all measurements. Notably, the SCT is commonly used in people with osteoarthritis of the hip and knee, as well as individuals undergoing TKA and THA, and has been shown to be reliable and responsive in these groups [52,53].

The 10 MWT assesses the participants’ walking speed in meters per second over a distance of 10 m, providing insight into participants’ gait, vestibular function and functional mobility. The measurement protocol for the self-paced 10 MWT as described by Hollman et al. [54] was used in this study. The time of 10 MWT was recorded for (1) subjects’ comfortable/normal self-paced walking speed, and (2) subjects’ fast self-paced walking speed. For fast 10 MWT, the command “walk as quickly as possible but in a safe manner” was used. Three trials under each condition—normal and fast—were performed. Velocity was calculated by dividing 10 m by the average time (s) for each condition. Participants were allowed to use their walking aid if needed. Although common in THA and TKA rehabilitation, no information on its reliability and responsiveness within the population could be obtained. However, Hollman et al. [54] showed excellent reliability in patients with hip fractures.

WOMAC is a self-reported questionnaire comprising 24 items (5 for pain, 2 for stiffness, 17 for functional limitations). Respondents rate each item on a scale from 1 (i.e., best) to 10 (i.e., worst). To perform statistical calculations with relative values, the cumulative score is divided by 24. WOMAC is considered a reliable and valid tool that can be used to assess the satisfaction of osteoarthritis patients after undergoing hip or knee arthroplasty [55].

HAQ-DI is a generic self-reported functional status measure. It is one of the most frequently used functional status assessments [56]. Scoring and HAQ-DI calculations were performed as described by Bruce and Fries [57]. HAQ-DI was shown to have good construct validity, internal consistency and reliability but limited responsiveness in people with general osteoarthritis by Cuperus et al. [58].

### 2.3. Statistical Analysis

Responsiveness is assessed by evaluating the relationship between the changes in scores of ID and other performance-based (TUG, SCT, 10 MWT) and self-reported (WOMAC, HAQ-DI) measures of PF from T0 (pre-surgery) to T3 (10 weeks post-surgery), through analysis of correlation, as recommended by García de Yébenes Prous et al. [59] and Terwee [36]. By formulating an explicit hypothesis on the expected direction and magnitude of correlations of changes, subsequent confirmation is made possible, data interpretation is enhanced and risk of bias is reduced [36]. For better data interpretability, we also estimated the test–retest reliability from T2 to T3 and the concordance of measurement instruments at T2 and T3, using Pearson’s Correlation Coefficient. Change scores for each measure are calculated as the difference, Delta (Δ), of the absolute score at T0 and T3 (Δ = T0 − T3). Furthermore, effect sizes of changes from T0 to T3 for each measure are reported as Cohens *d* with their according significance of difference *p* (* *p* ≤ 0.05, ** *p* ≤ 0.01, *** *p* ≤ 0.001, (*) *p* ≤ 0.1).

We expect the subject’s PF status to improve over the course of the study period. To show improvement in the parameters, the peak torque of ID and gait speed of 10 MWT will need to increase, and TUG time, SCT time, WOMAC score and HAQ-DI score will need to decrease. Therefore, we hypothesize the following correlations of change scores (Table 3).

The magnitude of the relationship for Pearson’s Correlation Coefficient is interpreted according to Hinkle et al. [60] (*r* < 0.5 low correlation, *r* = 0.5 to 0.7 moderate correlation, *r* > 0.7 high correlation). The correlation of change scores is evaluated for each of the eight PT parameters of ID separately. An ID parameter is considered responsive if direction and magnitude (Table 3) in three of the five hypotheses of expected change are established. We consider an error margin of *r* = |0.05| for the expected magnitude of the relationship eligible. IBMs SPSS version 28 software is used for statistical analysis.

### 2.4. Sample Size Estimation

Based on the hypothesized responsiveness by examining changes from T0 (before surgery) to T3 (10 weeks after surgery), the magnitude of the relationship between changes in muscle strength (ID) and other outcome measures of PF (TUG, SCT, 10 MWT, WOMAC, HAQ-DI), the Pearson correlation coefficient (*r*) should be greater than 0.5. Cohen [61] recommends a sample size of 22 test objects (*n* = 22) to statistically validate a moderate-to-high correlation (*r* = 0.5) with a significance level of α = 0.05 (one-sided) and a power of 1 − β = 0.80. With a sample size of *n* = 20, the post hoc calculation indicates an achieved power of 0.76 [62].

## 3. Results

### 3.1. Descriptive Analysis of Changes

None of the isokinetic PT parameters for the involved or uninvolved leg showed a significant difference between the pre-surgery and ten weeks post-surgery measurements (Table 4). In all other performance-based PF measures, statistically significant differences between T0 and T3 were observed, with effect sizes ranging from 0.42 to 0.92. The highest effect sizes were seen in TUG and normal 10 MWT (0.92 and 0.73, respectively). Significant changes were also seen in the self-reported PF measures HAQ-DI and WOMAC (all dimensions) with total WOMAC and WOMAC PF scores showing the highest overall effect size (1.05 and 1.04, respectively). We also investigated the alternative muscle performance parameter of total work accomplished throughout the entire set of isokinetic measurements. Total work done (TWD) showed a significant increase for 60°/s extension in both the involved and uninvolved leg as well as in 60°/s flexion in the involved side, with Cohens *d* ranging from 0.39 to 0.54 (*p* < 0.05). Significant differences between the operated and the uninvolved leg were seen for all PT parameters at both pre-surgery and post-surgery measurements (effect sizes ranged from *d* = 0.68 to *d* = 1.28).

### 3.2. Correlation of PF Measures

Analysis of test–retest reliability showed consistently high to very high positive correlations across all PF measures for absolute values from T2 to T3, with Pearson’s correlation coefficient ranging from *r* = 0.85 to *r* = 0.96 (*p* < 0.01). The exception was HAQ-DI, which demonstrated a moderate positive correlation of r = 0.67 (*p* < 0.01).

Concordance between PF measures at T2 ranged from |*r*| = 0.52 (*p* < 0.05) to |*r*| = 0.89 (*p* < 0.01), whereas the lowest correlations were experienced between WOMAC and 10 MWT and the highest between 10 MWT and TUG. At T3, concordance ranged from |*r*| = 0.47 (*p* = 0.57) to |*r*| = 0.90 (*p* < 0.01), whereas the lowest was between WOMAC and TUG and the highest, as with T2, between 10 MWT and TUG.

The correlation of changes from T2 to T3 between PF measures shows moderate to high positive correlations between all WOMAC dimensions (*r* = 0.60, *p* < 0.01 to *r* = 0.99, *p* < 0.001) but no significant correlation of any WOMAC dimension with any other PF measure (Table 5). In HAQ-DI, no significant correlation with any other PF measure was observed. Moderate negative correlations between SCT and 10 MWT at normal speed and fast speed (*r* = −0.56 and *r* = −0.62, respectively, *p* < 0.01) were detected. TUG showed a significant positive correlation with SCT (*r* = 0.45, *p* < 0.05).

### 3.3. Correlation of Isokinetic Peak Torque Parameters

Test–retest reliability for isokinetic PT parameters between T2 and T3 exhibited consistently high to very high positive correlations; Pearson’s correlation coefficients ranged from *r* = 0.83 to *r* = 0.97 (*p* < 0.01).

Concordance between isokinetic PT parameters at T2 ranged from moderate to very high (*r* = 0.51 (*p* < 0.05) to *r* = 0.96 (*p* < 0.01)), except for extension of the involved side at 180°/s, which experienced an insignificant low correlation with all flexion parameters (*r* = 0.36 to *r* = 0.48). At T3, concordance ranged from *r* = 0.43 (*p* < 0.1) to *r* = 0.95 (*p* < 0.01), whereas the lowest was between flexion of the involved leg and extension of the uninvolved leg at 60°/s.

The correlation analysis of changes within the different isokinetic PT parameters showed different patterns within the variables (Table 6). Notably, at a speed of 60°/s, there was a moderate positive correlation (*r* = 0.57, *p* < 0.001) between PT extension of the operated leg and PT extension of the uninvolved leg, as well as a moderate positive correlation (*r* = 0.50, *p* < 0.05) of PT flexion of the operated leg and PT flexion of the uninvolved leg. Conversely, no significant correlations between flexion and extension variables were observed at 60°/s. Similar trends were observed at 180°/s, with significant positive correlations between extension of the operated leg and extension of the uninvolved leg (*r* = 0.72, *p* < 0.001) and flexion of the operated leg and flexion of the uninvolved leg (*r* = 0.56, *p* < 0.05). Correlations between the two velocities were also observed in a corresponding pattern, with moderate to strong positive correlations between extension parameters at 60°/s and 180°/s (*r*-values ranging from 0.49 to 0.77) as well as moderate to strong positive correlations between flexion parameters at 60°/s and 180°/s (*r*-values ranging from 0.47 to 0.73).

### 3.4. Responsiveness

The results of the correlation analysis of changes in isokinetic PT and changes in the other PF measures are depicted in Table 7. For the operated side, associations were as follows: Knee extension at 60°/s shows a significant moderate positive correlation with 10 MWT normal speed (r = 0.45, *p* < 0.05) and a low positive correlation with fast speed (*r* = 0.26). Flexion at 60°/s exhibits a statistically significant moderate positive correlation with the HAQ-DI (*r* = 0.50, *p* < 0.05). Extension at 180°/s demonstrates significant positive correlations with 10 MWT at normal and fast speeds (*r* = 0.53 and *r* = 0.46, *p* < 0.05) and moderate negative correlations with HAQ-DI, SCT and TUG (*r* = −0.55, *r* = −0.48 and *r* = −0.47, *p* < 0.05). On the uninvolved side, extension at 180°/s shows significant moderate correlations with SCT and 10 MWT at fast and normal speeds (*r* = −0.48, *p* < 0.05; *r* = 0.56, *p* < 0.01; *r* = 0.54, *p* < 0.05). In contrast, flexion at 180°/s exhibits negligible correlations with all measures.

Comparing the observed correlations with the a priori defined expected direction and magnitude of the relationship of change scores reveals the following findings (Table 8). For knee extension of the operated side at 60°/s, accordance was found for HAQ-DI and 10 MWT normal speed within the eligible margin of error (*r* = |0.05|). Knee extension of the operated side at 180°/s showed agreement for HAQ-DI, SCT, TUG and 10 MWT (both speeds). Divergence in the expected direction of correlation with HAQ-DI was seen for flexion of the operated side in both angular velocities. In the uninvolved side, agreement was found for extension at 60°/s with HAQ-DI and for extension at 180°/s for HAQ-DI, SCT and 10 MWT at fast and normal speeds. No match of expected and observed correlations was seen in the change scores of knee flexion for both sides and both angular velocities. As we defined that an ID parameter is considered responsive if direction and magnitude in three of the five hypotheses of expected change are established, the only parameter that appears to be responsive by correlation analysis is PT of knee extension of the operated side at 180°/s.

## 4. Discussion

The aim of this study was to analyze the responsiveness of isokinetic dynamometry in the construct of PF in patients after hip and knee arthroplasty. We hypothesized that if the ID is responsive in the construct of PF, we would see moderate to high positive correlations between changes in isokinetic peak torque and changes in 10 MWT, moderate to high negative correlations between changes in ID and changes in TUG, SCT and WOMAC and low to moderate negative correlations between ID and HAQ-DI. We considered responsiveness to be established if the observed direction and magnitude of correlation in change scores were coherent with at least three of the five hypotheses of expected change defined in Table 3.

Our results indicate that only one of the eight tested ID parameters demonstrated responsiveness to change in the construct of PF. Specifically, the isokinetic peak torque of knee extension at 180°/s of the operated leg exhibited coherence in the observed and expected direction and magnitude of correlation in change scores for four of the five PF measures (Table 8). However, knee extension at 180°/s of the unoperated leg showed coherence with three hypotheses (HAQ-DI, 10 MWT and SCT) but did not fully meet the responsiveness criteria. Interestingly, none of the ID parameters correlated as expected with the WOMAC questionnaire, with low negative correlations observed for PT 60°/s Flexion operated, PT 60°/s Extension uninvolved and PT 60°/s Flexion uninvolved, demonstrating a tendency in the anticipated direction and magnitude of correlation. Surprisingly, discrepancies in the direction of the expected change relationship with HAQ-DI were observed for the ID parameters PT 60°/s Flexion operated and PT 180°/s Flexion operated. This suggests that in our sample, improvement in HAQ-DI is inversely related to improvement in knee flexion strength in the operated leg or vice versa. In summary, the results from our sample provide inconclusive evidence for the responsiveness of ID, as only one of the eight ID parameters demonstrated responsiveness, and some even exhibited discrepancies with the expected direction of correlation of change scores.

Our results differ from those of Holm et al. [63], who reported significant correlations between reduced fast-speed walking (10 MWT) and decreased knee-extension strength (*r* = 0.59; *p* = 0.003) acutely after surgery (1 week pre-surgery to post-surgery hospital discharge) in TKA patients, indicating that knee strength immediately after surgery might be disparately responsive in the construct of PF. However, responsiveness was not in the scope of Holm et al. [63].

The limited responsiveness exhibited might partially be explained by the lack of significant improvements in any of the isokinetic peak torque parameters we observed, while all other PF measures presented significant improvements between pre-surgery and 10 weeks post-surgery measurements, with moderate to high effect sizes. A possible limitation could be the last measurement, which took place 10 weeks post-surgery. That might not be long enough for the full effect of the surgery to happen. For many patients, symptoms and PF improve over time, even up to a year or more after the surgery. A shorter follow-up period may not capture the full extent of the improvement. We also exploratively looked into other parameters provided by the Biodex’s built-in results page. Total work accomplished throughout the entire set showed significant improvements for extension at 60°/s in both the involved and uninvolved legs as well as in flexion in the involved side at 60°/s, indicating improvement in some strength-related isokinetic parameters but not in maximum force. Our results are similar to the findings of Reardon et al. [64], who found a significant improvement in TUG (*p* = 0.007) but only minimal changes in the strength measurements of the quadriceps muscles of the operated leg over a 5-month postoperative period in THA patients. Research focusing on early postoperative strength after THA and TKA shows a different trend with a significant decrease in knee extensor strength and a significant worsening of functional performance measures (TUG, 10 MWT) from pre-surgery to hospital discharge [63,65].

To verify that the changes we observed were not due to measurement error or random variations, we estimated the test–retest reliability from T2 to T3. All measures exhibited good reliability with moderate to very high correlations of absolute values.

As we compared multiple peak torque measurements with multiple PF measures, the concordance of the measures became an influencing factor for responsiveness. If the chosen PF measures do not correspond well with each other, it becomes challenging to draw accurate and consistent conclusions on the responsiveness in the construct of PF. We saw moderate to very high concordance between PF measures at T2 and T3. WOMAC showed the least concordance with other PF measures, whereas the performance-based measures showed high concordance. This is also reflected in the correlation analysis of changes between the different functional measures, which indicates that they might change independently from each other (Table 6). Other researchers also found discrepancies when comparing PROMs to performance-based function in THA and TKA patients [13,66]. This lack of association between CROMs and PROMs of PF may be attributed to their assessment of partially divergent yet overlapping aspects of a patient’s abilities and impairments [67]. Our responsiveness results might have been affected by the choice to compare isokinetic strength not only with other performance-based measures but also with PROMs. However, the complementary use of PROMs and CROMs to evaluate PF in rehabilitation is suggested by multiple researchers [68,69].

Analyzing the concordance and correlation of changes within isokinetic peak torque measurements indicates independence of knee flexion and extension strength in our sample. However, we found a significant relationship between changes in strength of the operated leg and the unoperated leg as well as a significant relationship between the two angular velocities for knee extension as well as knee flexion. This is also reflected in the patterns exhibited in the responsiveness analysis with changes in flexion and extension parameters not experiencing the same correlations with changes in PF measures.

The main limitation of our study was the small and heterogeneous sample, encompassing both TKA and THA patients with an uneven gender ratio of three males to seventeen females (Table 1). However, our sample, consisting predominantly of females with an average age of 67 ± 8 years, is representative of rehabilitation patients with osteoarthritis after knee and hip arthroplasty in Austria [70]. Context and sample characteristics, such as gender or baseline values, play an important role in effect size statistics [69,71], like in sensitivity to treatment evaluation. In contrast, sample characteristics play a minor role in the correlative assessment of external responsiveness when using a criterion-oriented approach [59,72]. Our initial intention was to conduct separate responsiveness evaluations for subgroups. However, unforeseen technical issues and complications with the Biodex system prevented measurements in 17 instances, resulting in the exclusion of 11 participants (Figure 1). Rectifying the problem necessitated the expertise of a technician, causing a setback of several weeks. Unfortunately, this delay precluded any attempts to compensate for the reduced measurement capacity. Furthermore, the inability to measure strength shortly after surgery, as mandated by clinicians, hindered the assessment of strength development over the course of operation and rehabilitation separately. The progression of strength over time could have provided a more nuanced understanding of responsiveness, as the responsiveness at different time points, such as 1 week, 6 weeks and 10 weeks post-surgery, might vary.

A critical view also needs to be cast on the concept of responsiveness. There is a lack of consensus in the literature regarding responsiveness and appropriate measurement methods, with different statistics existing for various areas of responsiveness [59,73]. Transparency in the selected method is therefore crucial. The choice of responsiveness statistic depends on the characteristic of responsiveness and the type of expected change [59]. Simultaneously using multiple responsiveness measures in an article is not recommended, as it complicates comparison or even renders it impossible [72,74,75]. We chose to employ the approach outlined by Terwee [36], considering that it is the most recent and we agreed with the view that responsiveness is to be understood as validity of a change score. Consequently, our focus was on measuring longitudinal validity rather than relying on metrics indicating the magnitude of the treatment effect. Since the latter measures do not provide insights into the instrument’s efficacy in fulfilling its intended purpose, their primary utility lies in assessing the interpretability of changes in scores [36,73].

Assessing maximal strength following TKA and THA may not be the most appropriate metric. Research indicates that maximum muscle strength both before and after TKA is influenced by voluntary activation failure or arthrogenic muscle inhibition [76,77]. Recent studies propose shifting the focus from maximum force or peak torque measurements to the investigation of Rate of Force Development (RFD), as daily activities typically do not necessitate the exertion of maximum force from muscles [18,78]. Additionally, Maffiuletti et al. [18] demonstrated that RFD undergoes more significant changes post-TKA compared to maximum strength. We speculate that it may therefore serve as a more responsive measure and warrants further investigation.

While strength is commonly considered a key determinant of PF [16,17,18] and is associated both with self-reported and performance-based PF [78,79,80,81,82], our findings suggest that strength may not adequately capture changes in this construct. ID has inherent drawbacks, including the requirement for trained personnel, expensive equipment and the considerable time and effort needed for testing. System failures, as encountered in our case, render measurements impossible, necessitating a technician’s intervention and incurring additional time and financial costs for repairs.

The assessment of PF plays an essential role in the rehabilitation of THA and TKA patients. Recognized as an essential building block in overall quality of life, PF directly influences an individual’s ability to perform ADLs and IADLs, which are in turn vital for social, vocational and recreational participation [9]. Failing to meet the physical demands of these activities can considerably decrease an individual’s quality of life. Understanding the relationship between changes in PF and changes in muscle strength in patients undergoing TKA and THA is crucial for optimizing rehabilitation protocols and enhancing recovery outcomes. Both muscle strength and PF measures are used by clinicians and practitioners as indicators for improvements in rehabilitation after TKA and THA. Our analysis shows that isokinetic muscle strength experiences little to no relevant changes from pre-surgery to 10 weeks post-surgery and is not responsive to changes in PF. We therefore infer that isokinetic peak torque is not a valid indicator for improvements in rehabilitation post-TKA and -THA. Thus, it should not be used by clinicians as a measure of the effectiveness of treatment. We challenge the suitability of ID as a measurement tool in this context. However, it might still serve as a valuable training device and might still be used to identify and address specific deficits in muscle strength. As mentioned, peak torque might not be the most appropriate indicator of muscle performance in TKA and THA patients. Therefore, future research should also focus on other strength-related factors such as total work accomplished over the entire set or RFD, which might reflect PF more accurately as they seem to experience more relevant changes.

In light of the limited responsiveness observed, questions arise about the utility of isokinetic peak torque measurements in the early rehabilitation stages following TKA and THA. The practical constraints and high cost associated with ID, coupled with its inconclusive responsiveness, prompt queries about its role as the gold standard for strength assessment and its applicability in the clinical context of TKA and THA rehabilitation.

Recent research advocates that physical performance involves more than just muscle function, encompassing various other bodily organs and systems such as bones, balance, neurological inputs, cardiovascular aspects and motivation [83]. It indicates the need to comprehensively investigate how these factors and their interactions contribute to PF. It is important to point out that responsiveness is rarely studied in this context and therefore further research is needed to validate our results and improve our understanding of isokinetic dynamometry for the evaluation of PF after knee and hip replacement.

## 5. Conclusions

Our results indicate that isokinetic peak torque measurements of the knee hold limited responsiveness for the assessment of PF in TKA and THA rehabilitation. While proving to be reliable and concordant, isokinetic muscle strength of the knee flexors and extensors exhibit little to no changes after surgery and rehabilitation. We therefore conclude that it should not be used by clinicians as an indicator for improvements in PF or the effectiveness of rehabilitation. In the context of cost and practicality for clinical application, questions about its role as the gold standard for strength assessment and its usefulness in TKA and THA rehabilitation arise. The complexity of responsiveness evaluation and the intricacy of PF assessment make interpretation difficult and challenging. Therefore, additional research is needed to validate our results and improve our understanding of isokinetic dynamometry for the evaluation of PF after TKA and THA.

## Figures and Tables

**Table 1:** Key participant demographics.

	Total		Hip		Knee	
	Range	Mean ± SD	Range	Mean ± SD	Range	Mean ± SD
Age	[55.3–81.7]	66.6 ± 7.8	[58.8–81.7]	66.6 ± 7.9	[55.3–80.1]	66.5 ± 8.1
BMI	[22.5–43.9]	30.0 ± 5.5	[22.6–35.5]	28.7 ± 5.1	[22.5–43.9]	30.9 ± 5.8
Weight in kg	[62.8–118.1]	83.7 ± 14.8	[62.8–106.3]	85.2 ± 16.7	[64.8–118.1]	82.7 ± 14.7
Height in cm	[153–184]	167.2 ± 8.2	[157–184]	172.1 ± 8.8	[153–175]	163.9 ± 6.7
Physical activity *	[0.0–147.4]	42.2 ± 36.5	[4.6–78.0]	36.1 ± 27.7	[0.0–147.4]	46.7 ± 42.5
n (m/f)	20 (3/17)		8 (2/6)		12 (1/11)	

BMI—body mass index; m/f—male/female; SD—standard deviation. * Moderate to vigorous physical activity in minutes per average day using Daily Activity Behaviours Questionnaire (DABQ) [39].

**Table 2:** Measurement parameters of isokinetic dynamometry.

Angular Velocity		Operated Side	Uninvolved Side
60°/s	Flexion Extension	PT 60 flex op PT 60 ext op	PT 60 flex un PT 60 ext un
180°/s	Flexion Extension	PT 180 flex op PT 180 ext op	PT 180 flex un PT 180 ext un

PT—peak torque; flex—flexion; ext—extension; op—operated; un—uninvolved.

**Table 3:** Expected relationships between isokinetic dynamometry and physical function measures.

	Direction	Magnitude of Relationship
peak torque and gait speed (fast and normal)	+	moderate to high
peak torque and TUG time	−	moderate to high
peak torque and SCT time	−	moderate to high
peak torque and WOMAC scores (pain, stiffness, function)	−	moderate to high
peak torque and HAQ-DI score	−	low to moderate

HAQ-DI—HAQ-disability index; SCT—stair climbing test; +—positive correlation; −—negative correlation.

**Table 4:** Mean scores of physical function measures before and ten weeks after surgery.

Outcome	N	T0 Mean ± SD	T3 Mean ± SD	Δ	Cohens d (Significance)
higher scores indicate better functioning					
PT 60°/s Flexion operated leg (Nm)	20	32.74 ± 20.93	36.48 ± 19.92	3.74	0.38 (*)
PT 60°/s Extension operated leg (Nm)	20	67.73 ± 32.44	71.02 ± 28.48	3.29	0.21
PT 60°/s Flexion uninvolved leg (Nm)	20	44.51 ± 20.14	46.19 ± 16.79	1.68	0.17
PT 60°/s Extension uninvolved leg (Nm)	20	91.38 ± 31.37	95.41 ± 31.56	4.03	0.27
PT 180°/s Flexion operated leg (Nm)	20	27.16 ± 13.60	27.48 ± 13.28	0.32	0.04
PT 180°/s Extension operated leg (Nm)	20	50.79 ± 21.96	53.46 ± 20.72	2.67	0.20
PT 180°/s Flexion uninvolved leg (Nm)	20	33.68 ± 16.18	32.86 ± 14.69	0.82	0.09
PT 180°/s Extension uninvolved leg (Nm)	20	62.67 ± 22.05	64.72 ± 23.26	2.05	0.26
10 MWT fast (m/s)	20	1.46 ± 0.32	1.57 ± 0.39	0.11	0.56 *
10 MWT normal (m/s)	20	1.12 ± 0.20	1.20 ± 0.23	0.08	0.73 **
lower scores indicate better functioning					
TUG (s)	20	10.45 ± 2.53	9.05 ± 2.32	1.40	0.92 ***
SCT (s)	20	17.62 ± 8.00	14.83 ± 7.04	2.79	0.42 *
WOMAC pain	19	19.84 ± 11.52	11.79 ± 7.20	8.05	0.91 ***
WOMAC stiffness	19	9.58 ± 4.60	5.47 ± 3.32	4.11	0.94 ***
WOMAC PF	19	65.89 ± 39.82	39.26 ± 25.37	26.63	1.04 ***
WOMAC total	19	95.32 ± 54.24	57.05 ± 33.84	38.27	1.05 ***
HAQ-DI	18	0.86 ± 0.54	0.63 ± 0.44	0.23	0.56 *

* *p* ≤ 0.05; ** *p* ≤ 0.01; *** *p* ≤ 0.001 (*) *p* ≤ 0.1; SD—standard deviation; Δ—Delta = |T0 − T3|; 10 MWT—10 m walk test; HAQ-DI—HAQ-disability index; PT—peak torque; SCT—stair climbing test.

**Table 5:** Correlations of changes in physical function measures pre- to post-surgery.

Variable	WOMAC Pain	WOMAC Stiffness	WOMAC Function	WOMAC Total	HAQ-DI	SCT	10 MWT Normal	10 MWT Fast	TUG
WOMAC Pain	1.00	0.60 **	0.82 ***	0.87 ***	0.39	−0.04	0.11	−0.06	−0.06
WOMAC Stiffness		1.00	0.82 ***	0.85 ***	0.29	0.02	−0.13	−0.09	−0.16
WOMAC Function			1.00	0.99 ***	0.29	−0.24	0.18	0.13	−0.15
WOMAC Total				1.00	0.31	−0.17	0.15	0.09	−0.14
HAQ-DI					1.00	0.28	−0.31	−0.43	0.41
SCT						1.00	−0.56 **	−0.62 **	0.45 *
10 MWT normal							1.00	0.84 ***	−0.29
10 MWT fast								1.00	−0.35
TUG									1.00

* *p* ≤ 0.05; ** *p* ≤ 0.01; *** *p* ≤ 0.001; 10 MWT—10 m walk test; HAQ-DI—HAQ-disability index; SCT—stair climbing test.

**Table 6:** Correlations of changes in isokinetic peak torque parameters pre- to post-surgery.

Variable	PT 60 ext op	PT 60 flex op	PT 180 ext op	PT 180 flex op	PT 60 ext un	PT 60 flex un	PT 180 ext un	PT 180 flex un
PT 60 ext op	1.00	−0.13	0.72 ***	−0.09	0.57 ***	−0.28	0.61 ***	−0.24
PT 60 flex op		1.00	−0.35	0.47 *	−0.05	0.50 *	−0.02	0.50 *
PT 180 ext op			1.00	−0.07	0.49 *	−0.27	0.72 ***	−0.17
PT 180 flex op				1.00	0.23	0.55 *	0.41	0.56 *
PT 60 ext un					1.00	0.18	0.77 ***	0.00
PT 60 flex un						1.00	0.03	0.73 ***
PT 180 ext un							1.00	0.20
PT 180 flex un								1.00

* *p* ≤ 0.05; *** *p* ≤ 0.001; PT—peak torque; flex—flexion; ext—extension; op—operated; un—uninvolved.

**Table 7:** Correlations of changes in isokinetic strength and physical function measures.

		PT 60 ext op	PT 60 flex op	PT 180 ext op	PT 180 flex op	PT 60 ext un	PT 60 flex un	PT 180 ext un	PT 180 flex un
WOMAC	Pain	0.10	−0.25	−0.06	0.17	−0.21	−0.44 (*)	0.04	−0.16
	Stiffness	−0.23	−0.24	−0.14	0.00	−0.40	−0.29	−0.18	0.08
	Function	−0.05	−0.33	−0.03	0.17	−0.26	−0.41	0.06	−0.05
	Total	−0.05	−0.32	−0.05	0.16	−0.27	−0.41	0.04	−0.04
HAQ-DI		−0.37	0.50 *	−0.55 *	0.34	−0.31	0.10	−0.28	0.23
SCT		−0.31	0.10	−0.48 *	−0.14	−0.09	0.08	−0.48 *	−0.17
10 MWT	normal	0.45 *	−0.23	0.53 *	0.26	0.41	−0.07	0.54 *	−0.04
	fast	0.26	−0.11	0.46 *	0.41	0.35	0.18	0.56 **	0.21
TUG		−0.23	0.34	−0.47 *	−0.06	−0.13	−0.09	−0.33	−0.07

* *p* ≤ 0.05; ** *p* ≤ 0.01; (*) *p* = 0.1; █ Low correlation; █ Low correlation within margin of error *r* = |0.05|; █ Moderate correlation; █ Moderate correlation within margin of error *r* = |0.05|; 10 MWT—10 m walk test; HAQ-DI—HAQ-disability index; SCT—stair climbing test; PT—peak torque; flex—flexion; ext—extension; op—operated; un—uninvolved.

**Table 8:** Comparison of expected and observed relationship of change scores.

		PT 60 ext op	PT 60 flex op	PT 180 ext op	PT 180 flex op	PT 60 ext un	PT 60 flex un	PT 180 ext un	PT 180 flex un
WOMAC Pain, Stiffness, Function, Total									
HAQ-DI		✓	✗	✓	✗	✓		✓	
SCT				✓				✓	
10 MWT	normal	✓		✓				✓	
	fast			✓				✓	
TUG				✓					

✓ expected direction and magnitude of relationship observed; ✗ discrepancy of expected and observed direction; 10 MWT—10 m walk test; HAQ-DI—HAQ-disability index; SCT—stair climbing test; PT—peak torque; flex—flexion; ext—extension; op—operated; un—uninvolved.

## Data Availability

The datasets analyzed in this manuscript are not publicly available because of ethical and legal restrictions (data contain potentially identifying and sensitive patient information). If not already reported within this work, the authors may provide descriptive data on individual outcomes for the different measurement time points. Requests for access to anonymized datasets should be directed to the corresponding author.
